# Acceptability of Telehealth as the Default Modality for Multiple Sclerosis Care in Switzerland: Cross-Sectional Study

**DOI:** 10.2196/84447

**Published:** 2026-01-23

**Authors:** Sintieh Nchinda Ngek Ekongefeyin, Paola Daniore, Vasileios Nittas, Stefania Iaquinto, Enriqueta Vallejo-Yagüe, Christian P Kamm, Pasquale Calabrese, Claudia Baum, Claudio Gobbi, Chiara Zecca, Andrew Chan, Milo Puhan, Viktor von Wyl

**Affiliations:** 1 Institute for Implementation Science in Health Care, University of Zurich, Universitatstrasse 84, Zurich, 8006, Switzerland; 2 Digital Society Initiative, University of Zurich, Zurich, Switzerland; 3 Epidemiology, Biostatistics, and Prevention Institute, University of Zurich, Zurich, Switzerland; 4 Center for Digital Trust, Swiss Federal Institute of Technology Lausanne, Lausanne, Switzerland; 5 Institute of Primary Health Care (BIHAM), Faculty of Medicine, University of Bern, Bern, Switzerland; 6 Neurocenter, Luzerner Kantonsspital, Lucerne, Switzerland; 7 Department of Neurology, Inselspital, University Hospital Bern, University of Bern, Bern, Switzerland; 8 Neuropsychology and Behavioral Neurology Unit, Division of Cognitive and Molecular Neuroscience, University of Basel, Basel, Switzerland; 9 Rehaklinik Zihlschlacht AG, Neurological Rehabilitation Center, Part of the VAMED Group, Zihlschlacht, Switzerland; 10 Department of Neurology, Neurocenter of Southern Switzerland, Ospedale Regionale di Lugano, EOC, Lugano, Switzerland; 11 Faculty of Biomedical Sciences, Università della Svizzera Italiana, Lugano, Switzerland

**Keywords:** acceptance, chronic disease management, digital health, multiple sclerosis, patient preferences, telehealth as a default, telemedicine

## Abstract

**Background:**

Telehealth can improve access to care for people living with multiple sclerosis (MS), but information on its acceptance is limited in Switzerland.

**Objective:**

This study aimed to determine the proportion of people living with MS willing to accept telehealth as a new default and the factors associated with their acceptance.

**Methods:**

We conducted a cross-sectional analysis using survey data from the Swiss Multiple Sclerosis Registry. We defined “telehealth as a default” as a health care model where remote consultations (telephone and/or video calls) are the primary mode of interaction between patients and their physicians, with in-person visits based on clinical necessity. Multivariable logistic regression was performed to evaluate the association between telehealth acceptance and sociodemographic and health-related factors. Telehealth acceptance was described in relation to 3 survey variables that mirrored key constructs from the Non-Adoption, Abandonment, Scale-Up, Spread, and Sustainability (NASSS) framework. The variables were digital communication preferences, internet use for health provider searches, and experience with telemedicine.

**Results:**

Among 427 respondents, 15.5% (66/427) reported a willingness to accept telehealth as their default. In this group, only 21.2% (14/66) had experience using telemedicine. A descriptive analysis of our 3 NASSS-derived key constructs showed that among the 78.5% (335/427) respondents who generally agreed to digital access to health data, only 17.0% (57/335) accepted telehealth as a default. Notably, 30.7% (129/427) of participants stated a wish for support for using devices or the internet. Among those 129 individuals, 17.1% (22/129) were willing to accept telehealth as a default. Of the 89 people with prior telehealth experience, 15.7% (14/89) were willing to accept telehealth. In multivariable analysis, digital communication with health care providers (adjusted odds ratio [aOR] 14.56, 95% CI 6.18‐39.04; *P*<.001*),* current internet use for health care provider search (aOR 7.78, 95% CI 1.34‐45.32; *P*=.021)*,* and a secondary progressive MS diagnosis (aOR 0.22, 95% CI 0.05‐0.72; *P*=.021) were independently associated with accepting telehealth as a default.

**Conclusions:**

Our findings suggest a low acceptance of telehealth as a default among people living with MS in Switzerland. While our 3 postulated NASSS-derived key constructs were not associated with telehealth acceptance, we noted additional behavioral factors, including previous digital communication with health care providers and using the internet to search for health care provider information, which were associated with telehealth acceptance. Moreover, advanced disease states like secondary progressive MS were negatively associated with telehealth acceptance. Thus, telehealth as a default will be most acceptable in people living with MS who already use the internet for their health, and those with less severe disease. Future research should explore provider perspectives and evaluate long-term strategies for the acceptance of telehealth in MS care.

## Introduction

Telehealth, the use of information and communication technology to provide remote health care, is a relatively novel approach in chronic disease management [1]. If widely adopted, it has the potential to improve access, continuity, and quality of care while reducing costs for populations with chronic diseases [2,3]. Evidence of patient benefits through its application in chronic disease care is promising. When used among people with cardiovascular disease (CVD), half of them had improved CVD risk scores [4]. When used among people living with diabetes, the quality of care was comparable to standard care while improving access and reducing the cost of care [5]. Among people living with cancer, it improved the quality of life and psychological well-being [6]. The COVID-19 pandemic also accelerated the use of telehealth in patient care, demonstrating its feasibility and acceptability for both patients and clinicians in chronic disease care, including multiple sclerosis (MS) [7,8].

In MS care, telehealth has been used for follow-up consultations, cognitive assessments, rehabilitation, and the management of symptoms that may limit mobility or access to specialist care. Altman et al [9] reported that both people living with MS and health care providers had a high level of satisfaction with telehealth. A review reported that people living with MS and their health care providers generally viewed telehealth as an acceptable and effective alternative to traditional in-person visits [10]. Also, telehealth has been reported to improve self-management [11,12], anxiety and depression [13-15], and activities of daily living in people living with MS [16]. Finally, no adverse events were reported with telerehabilitation for people living with MS [17].

The successful implementation of telehealth in MS and other chronic diseases is influenced by a range of enablers and barriers. Enablers include the perceived value proposition of the technology, such as improved access, convenience, and patient outcomes, alongside supportive organizational structures and positive attitudes among both patients and clinicians [18-20]. Conversely, barriers often arise from the severity of the disease, organizational complexity, technological challenges, digital literacy gaps, and concerns regarding data security or the adequacy of remote clinical assessments [16,21-23].

To systematically assess enabling and inhibiting factors, several frameworks have been proposed [24-26]. A widely used framework is the Non-Adoption, Abandonment, Scale-Up, Spread, and Sustainability (NASSS) framework [27]. The NASSS framework provides a comprehensive approach to understanding the adoption and sustained use of technologies in health care and has been used for a wide range of technologies and disease areas [27], including the use of telehealth in chronic care for conditions like CVDs, cancers, and neurological disorders [26]. The framework evaluates the complexity of technology acceptance within 7 constructs included in NASSS, which aim to identify and address factors influencing technology acceptance, anticipate barriers to implementation, and design interventions that are more likely to achieve scale-up and sustainability.

In MS care, few studies examine under what conditions people living with MS would accept telehealth as a default. In our study, we define “telehealth as a default” as a health care delivery model where remote consultations are the primary mode of interaction between patients and their physicians, with in-person visits occurring based on clinical necessity [28]. Previous studies report high patient and provider satisfaction [29], variable confidence in using telehealth in MS care [19], barriers limiting telehealth adoption [23]; but fail to report what conditions would enable or inhibit telehealth as a default among people living with MS.

Overall, many preconditions for successful telehealth implementation in Switzerland should be in place, including the presumed (and partially demonstrated) advantages of telehealth, the generally positive health-seeking attitude of people living with MS in Switzerland [30,31], and the push from global and national authorities for digital infrastructure to reduce diagnostic and therapeutic delays [1,32,33]. However, it remains unclear to what extent the Swiss population of people living with MS is capable and willing to engage in telehealth as a default mode for routine consultations. Therefore, our research seeks to assess the proportion of people living with MS in Switzerland potentially willing to accept telehealth as a default and the factors influencing their acceptance. Our findings are contextualized using the NASSS framework [34] and will provide information on how to target people living with MS with telehealth and consequently improve the quality of care for people living with MS.

## Methods

### Study Design and Setting

This study was a nested cross-sectional analysis within the Swiss Multiple Sclerosis Registry (SMSR), an ongoing observational cohort initiated in 2016. The SMSR recruits adults with MS residing or receiving treatment in Switzerland. Participation is voluntary and requires written informed consent, alongside confirmation of MS or clinically isolated syndrome (CIS) diagnosis. Upon enrollment, participants complete a baseline questionnaire and are invited to provide self-reported data via follow-up questionnaires approximately twice a year, available both online and in paper format.

### Ethical Considerations

Ethical approval for the SMSR was granted by the ethics committee of the Canton of Zurich (PB–2016–00894; BASEC-NR 2019–01027). Further details on the SMSR have been previously published [35,36]. All participants had previously provided informed consent to be contacted and the use of their data for research purposes, including secondary analyses. Participation in this survey was voluntary, and individuals could decline without consequences. All data provided to the study team were fully anonymized. To protect participants’ privacy and confidentiality, only anonymized data were used for this analysis, and results are reported in aggregate such that no individual participant can be identified. Participants received no compensation for completing the survey.

For this analysis, we used data from an anonymous, complementary online survey conducted between April 2023 and June 2023. This survey is a follow-up investigation of an earlier study on patterns of digital device use and digital literacy that was launched in October 2020 [37]. The 2023 survey used for this analysis was conducted online, in German, and offered independently of routine SMSR follow-ups (n=1720 eligible; 431 respondents; Multimedia Appendix 1). The rationale for offering the follow-up survey to a limited subsample had several reasons. First, we aimed to inform ongoing debates on telehealth and electronic health records promptly and therefore decided to offer the survey only in one language and online, also to facilitate data analysis. Second, our decision to offer the survey online and anonymously was also designed to attract inactive participants (ie, those who had not completed surveys in the past 2 years but remained in the cohort). Including these participants was intended to mitigate nonresponse bias and to capture perspectives from people living with MS who may be less engaged with research or digital tools. Third, the well-described SMSR source population and the existence of results from the 2020 study, which was offered to the full SMSR study population in all languages and participation modes (online and paper-pencil), enabled us to examine the generalizability and projections of key findings based on the limited, second survey (Multimedia Appendix 1).

### Using the NASSS Framework for Hypothesis Development and Interpretation of Findings

The NASSS framework provides a structured approach to understanding the facilitators and barriers to technology uptake [34]. In our study, we used the NASSS framework to generate hypotheses, given the available data, for which factors may determine the acceptance of telehealth as a default by people living with MS in Switzerland. It comprises 7 domains: *the condition*—the nature of the health condition; *the technology*—the technology itself (eg, usability and reliability); *the value proposition*—the value of the technology to health care stakeholders; *the adopters*—the individuals expected to adopt the technology (patients, clinicians, and carers); *the organization*—the health care organizational context; *the wider system*—the wider system including policy and regulatory factors; and *embedding over time*—how the technology and its use may evolve through processes of embedding and adaptation. To describe the complexity of technology acceptance in the aforementioned domains, the NASSS framework defines a “simple technology” as one that has few components and its successful implementation is predictable, a “complicated technology” as one that has multiple components and its success is largely predictable, and a “complex technology” as one that has numerous interacting components with limited chances of succeeding and hence, less likely to be adopted [27]. Systematic reviews applying the NASSS framework have identified that barriers are most frequently encountered within the domains of organization and adopter system, while enablers are linked to clear value propositions and alignment with patient needs [26,34,38].

### Main Outcomes

The primary study outcome was the proportion of participants who accepted telehealth as a default method for consultations. The acceptance of telehealth as a default was assessed using the following question: “Agreement to Digital Default (Would you find it beneficial for your medical care if medical consultations were through telemedicine?),” collected as a Likert scale from 0 (completely disagree) to 4 (completely agree). For further analysis, a binary variable was created where “Yes” included Likert levels (3,4) and “No” included levels (0,1,2). When describing the prevalence of acceptance in the population, we refer to the observed proportion as “low acceptance” descriptively; we did not define formal quantitative thresholds for low versus high acceptance.

### Main Explanatory Variables of Interest

We hypothesized 3 main drivers of telehealth acceptance that are included in our data: (1) knowledge of and prior experience with telehealth, (2) general openness toward digital health tools and electronic health records, and (3) needs and wishes for digital support.

Knowledge of and experience with telehealth was assessed using the following question: *“*Do you have knowledge of telemedicine offerings, and have you already gathered experiences with telemedicine?*”* Answer options were *“*neither knowledge of nor experience with telemedicine,*” “*knowledge of but no experience with telemedicine,*”* or *“*knowledge of and experience with telemedicine.*”*

Openness for use of other digital health tools was assessed through items on willingness to accept other digital health services (eg, file sharing, health data use, and provider communication) and experience with telemedicine. Each variable was collected as a Likert scale ranging from 0 (completely disagree with digital default) to 4 (completely agree with specific digital default).

Digital support needs were assessed with two items: (1) *“*Do you require support in using digital devices (eg, smartphones, laptops)?” and (2) *“*Do you need assistance with internet-related tasks (eg, security, navigation, program management)?*”* Each was rated on a Likert scale from 0 (no support needed) to 2 (extensive support needed). Participants reporting moderate (1) or extensive support (2) needs for both items were classified as requiring digital support.

### Other Explanatory Variables

Covariates included age (categorized as 18‐30, 31‐40, 41‐50, 51‐60, 61‐70, >70 y), sex (male or female), MS type (CIS or relapsing-remitting MS, primary progressive MS [PPMS], secondary progressive MS [SPMS]), and frequency of internet use for health-related purposes (eg, appointment scheduling and provider communication). Prior or current telemedicine use was included as a binary variable. Additional covariates included concerns related to internet use (eg, data security and information reliability). Internet use frequency was categorized as never, less than monthly, monthly, weekly, daily, or several times daily. A composite “use score” (range 0‐5) was calculated by summing the number of online activities performed at least monthly.

### Statistical Analysis

We first described the study population using MS type, sociodemographic characteristics, and experience with telemedicine. We then compared key variables based on previous telehealth experience (Multimedia Appendix 2) and openness to default telehealth consultations (see Table 1). In this study, a complete case analysis was undertaken. We excluded persons where key variables were missing from the analysis, leading to the exclusion of 4 participants.

**Table 1.** Comparison of preferences by default telehealth consultation status (N=427).

Variables	Overall, n (%)	Telehealth consultation as default =no (n=361), n (%)	Telehealth consultation as default=yes (n=66), n (%)
Descriptive variables			
* *Age group (y)			
18‐30	11 (2.6)	9 (2.5)	2 (3.0)
31‐40	56 (13.1)	48 (13.3)	8 (12.1)
41‐50	108 (25.3)	90 (24.9)	18 (27.3)
51‐60	143 (33.5)	121 (33.5)	22 (33.3)
61‐70	81 (19.0)	73 (20.2)	8 (12.1)
>70	28 (6.6)	20 (5.5)	8 (12.1)
Sex			
Female	298 (69.8)	256 (70.9)	42 (63.6)
Male	129 (30.2)	105 (29.1)	24 (36.4)
MS^a^ types			
CIS^b^ and RRMS^c^	285 (66.7)	240 (66.5)	45 (68.2)
PPMS^d^	51 (11.9)	44 (12.2)	7 (10.6)
SPMS^e^	91 (21.3)	77 (21.3)	14 (21.2)
Hypothesis 1: Have you ever used a telemedicine service?			
No knowledge, no experience	169 (39.6)	143 (39.6)	26 (39.4)
Knowledge, no experience	169 (39.6)	143 (39.6)	26(39.4)
Knowledge, experience	89 (20.9)	75 (20.8)	14 (21.2)
Hypothesis 2: acceptability to digital default (Would you find any of the following functionalities beneficial for your medical care?)			
Sell your health data (agree)	102 (24.2)	72 (20.2)	30 (45.5)
Default digital access to health data (agree)	335 (78.5)	277 (76.7)	58 (87.9)
Default digital document exchange (agree)	236 (55.7)	187 (52.2)	49 (74.2)
Default digital communication with a health care provider (agree)	167 (39.1)	114 (31.6)	53 (80.3)
Hypothesis 3: In which areas do you wish for more support in the “digital” world?			
Need support when using the internet (yes)	104 (24.8)	85 (24.0)	19 (28.8)
Need support when using hardware (yes)	109 (25.8)	86 (24.2)	23 (34.8)
Needs support for both	129 (30.2)	105 (29.7)	24 (36.4)
Other potentially explanatory variables			
* *Which concerns do you have related to internet use?			
Data security (agree)	271 (64.2)	235 (66.0)	36 (54.5)
Reliability of information “invalid data” (agree)	252 (60.0)	220 (62.1)	32 (48.5)
Isolation from real life (agree)	97 (23.1)	80 (22.6)	17 (25.8)
Health effects of internet use (agree)	70 (16.6)	54 (15.2)	16 (24.2)
For which activity do you go online when related to the topic of medicine and health (components for use score)? Percentage with at least 1 monthly activity			
Exchange with other patients	41 (10.5)	33 (9.9)	8 (13.6)
Self-tracking	43 (11.2)	32 (9.7)	11 (20.0)
Communication with a health care provider	57 (14.0)	44 (12.8)	13 (21.0)
Health care provider search	30 (7.5)	21 (6.2)	9 (15.0)
MS information search	195 (48.6)	157 (46.3)	38 (61.3)
Health information search	249 (62.7)	209 (61.5)	40 (70.2)
Private appointments (yes)	250 (58.5)	208 (57.6)	42 (63.6)
Professional appointments (yes)	188 (44.0)	157 (43.5)	31 (47.0)
All appointment types (yes)	299 (70.0)	250 (69.3)	49 (74.2)
How often do you use electronic devices (at least monthly)			
Frequency of smartwatch use	57 (18.6)	48 (18.0)	9 (22.0)
Frequency of smartphone use	365 (95.3)	307 (94.8)	58 (98.3)
Frequency of tablet use	146 (45.8)	126 (45.7)	20 (46.5)
Frequency of PC use	294 (89.1)	246 (88.8)	48 (90.6)

a MS: multiple sclerosis.

b CIS: clinically isolated syndrome.

c RRMS: relapsing-remitting multiple sclerosis.

d PPMS: primary progressive multiple sclerosis.

e SPMS: secondary progressive multiple sclerosis.

Univariable logistic regression models were used to identify factors associated with willingness to accept default telehealth consultations (Multimedia Appendix 3). All prespecified variables relating to the use of digital tools and attitudes toward digitalization were included in the full multivariable logistic regression model, which also included predefined adjustments for potential confounders (age, sex, MS type, and telemedicine experience). A 2-sided *P* value <.05 was considered statistically significant. All analyses were conducted using R (version 4.4.2; R Foundation for Statistical Computing).

### Reporting Framework

This paper was written following the STROBE (Strengthening the Reporting of Observational Studies in Epidemiology) guidelines (Checklist 1) for cross-sectional studies [39].

## Results

### Sociodemographic Data

Of 431 survey respondents, 4 had missing information regarding age. Therefore, a total of 427 participants with complete data were included in the analysis. The study sample consisted of 30.2% (129/427) men and 69.8% (298/427) women, with 41% (175/427) of participants aged between 18 and 50 years. Of the respondents, 66.7% (285/427) reported having relapsing-remitting MS or CIS, while 11.9% (51/427) had PPMS and 21.3% (91/427) SPMS. A total of 20.9% (89/427) reported previous experience with telemedicine consultations (Table 2).

**Table 2.** Characteristics of participants (N=427).

Variable	All participants, n (%)
Age group (y)	
18‐30	11 (2.6)
31‐40	56 (13.1)
41‐50	108 (25.3)
51‐60	143 (33.5)
61‐70	81 (19)
70+	28 (6.6)
Sex	
Female	298 (69.8)
Male	129 (30.2)
MS^a^ types	
CIS^b^ and RRMS^c^	285 (66.7)
PPMS^d^	51 (11.9)
SPMS^e^	91 (21.3)
Experience or knowledge with telemedicine	
No experience or knowledge	169 (39.6)
Telemedicine knowledge, no experience	169 (39.6)
Telemedicine knowledge and experience	89 (20.9)

a MS: multiple sclerosis.

b CIS: clinically isolated syndrome.

c RRMS: relapsing-remitting multiple sclerosis.

d PPMS: primary progressive multiple sclerosis.

e SPMS: secondary progressive multiple sclerosis.

### Acceptance of Telehealth as a Default Method of Consultation

A total of 66 out of 427 (15.5%) participants were open to a default telehealth consultation (Table 1). Among this group, 21.2% (14/66) had previous telemedicine experience. Although 78.5% (335/427) agreed to default digital access to health data, only 17.3 (58/335) agreed to default to telehealth. Of the 30.2% (129/427) participants needing hardware and software support, 81.4% (105/129) did not agree to telehealth as a default. Older age groups greater than or equal to 60 years 90.1% (73/81, ) and those concerned with data security 86.7% (235/271, ) and information validity 87.3%(220/252, ) rarely accepted telehealth as a default.

### Factors Associated With Acceptance of Telehealth as a Default

#### Multivariable Regression

In the multivariable regression model (Table 3), default communication with health care providers (adjusted odds ratio [aOR] 14.56, 95% CI 6.18‐39.04; *P*<.001), internet use for health care provider search (aOR 7.78, 95% CI 1.34‐45.32; *P*=.021) and a SPMS diagnosis (aOR 0.22, 95% CI 0.05‐0.72; *P*=.021) were independently associated with an agreement to default telehealth consultation. Other factors, such as concerns about invalid information, data security, and telemedicine experience, did not show significant associations when adjusted for other predictors. The multivariable model showed low multicollinearity (variance inflation factor <1.5), suggesting that the predictors are not highly correlated with each other and that the regression coefficients can be reliably interpreted.

**Table 3.** Multivariable logistic regression for telehealth consultations.

Variable	aOR^a^ (95% CI)			*P* value
Descriptive variables				
Sex (male)	0.88 (0.35-2.11)			.778
Age (y)				.271^b^
18‐30 (reference)	—^c^			
31‐40	0.49 (0.07-4.60)			
41‐50	0.60 (0.10-5.12)			
51‐60	0.67 (0.10-5.89)			
61‐70	0.55 (0.07-5.41)			
>70	4.15 (0.40-54.92)			
MS^e^ type				.041^d^
MS type: CIS^f^ or RRMS^g^ (reference)	—			
MS type: PPMS^h^	0.67 (0.17-2.24)			
MS type: SPMS^i^	0.22 (0.05-0.72)			
Hypothesis 1: Have you ever used a telemedicine service?				
Telemedicine experience (yes or no; reference)	0.94 (0.37-2.23)			.888
Hypothesis 2: agreement to digital default (Would you find any of the following functionalities beneficial for your medical care?)				
Default digital communication with HCP^j^	14.56 (6.18-39.04)			<.001
Default digital access to health data	0.65 (0.19-2.34)			.492
Default digital document exchange	1.60 (0.66-4.15)			.311
Hypothesis 3: In which areas do you wish for more support in the “digital” world?				
Needs support (yes or no)	1.98 (0.84-4.60)			.112
Other explanatory variables				
Concern about invalid information	0.96 (0.42-2.20)			.920
Concerned about data security	0.85 (0.37-1.97)			.702
Uses the internet for health info search	0.86 (0.37-2.05)			.737
Uses internet for MS info search	1.68 (0.73-3.93)			.225
Uses the internet for HCP search	7.78 (1.34-45.32)			.021
Uses the internet for communication with an HCP	0.62 (0.19-1.82)			.409
Uses the internet for self-tracking	1.38 (0.45-3.95)			.560
Uses the internet for patient exchange	1.02 (0.28-3.21)			.980

a aOR: adjusted odds ratio.

b Likelihood ratio test: *χ*²_5_=5.49; *P*=.36.

c Not applicable.

d Likelihood ratio test: *χ*²_2_=6.44; *P*=.040.

e MS: multiple sclerosis.

f CIS: clinically isolated syndrome.

g RRMS: relapsing-remitting multiple sclerosis.

h PPMS: primary progressive multiple sclerosis.

i SPMS: secondary progressive multiple sclerosis.

j HCP: health care professional.

## Discussion

### Summary of Findings

This cross-sectional study determined the proportion of people living with MS who are willing to accept telehealth as a default and the factors influencing their acceptance. We found that only 66 out of 427 (15.5%) participants were willing to accept telehealth as a default, which was positively associated with a general openness for digital communication with health care providers or using the internet for health care provider searches. We also found a negative association of telehealth acceptance with having SPMS.

### The Proportion of People Living With MS Who Accepted Telehealth as a Default

In our study, less than one-fifth of people living with MS accepted telehealth as a default. However, this finding contrasts with other studies reporting over 70% acceptance of telehealth among people living with MS compared to in-person visits [23,29,40]. These earlier studies examined acceptance and satisfaction with telehealth during the COVID-19 pandemic, a period in which the extensive use of telemedicine ensured continuity of care, which probably contributed to increased acceptance. Hence, the difference in rates of acceptance might be explained by the forced global shift to digital services to curb the pandemic. Also, these earlier investigations did not specifically assess accepting telehealth as a default. Our low acceptance rate could also be due to the complexity of MS and factors like comorbidities and the older age of our study sample [41]. MS severity is often linked with more physical impairments, comorbidities, and aging [42]. In our study, persons with PPMS and SPMS, as well as persons aged over 60 years, were overrepresented compared with the projected population of people living with MS in Switzerland (Multimedia Appendix 1). These sample differences may explain the low percentage of telehealth acceptance as a default. Indeed, studies have repeatedly shown that people aged above 60 years are least accepting telehealth as a default; that corroborates with research on technology acceptance in older populations [23,27,43-45]. Nevertheless, some research highlights that older adults may still be receptive to telehealth when it addresses access barriers, reduces the burden of travel, or is perceived as enhancing continuity of care [46,47]; hence, addressing these preconditions may increase acceptance of telehealth.

The contrast between the high acceptance of default digital access to health data (78.5%) and the low acceptance of telehealth as default MS care (17.1%) suggests that people living with MS may favor asynchronous over synchronous digital modalities. Asynchronous tools such as secure messaging, patient portals, and digital care paths enable patients to access information, contact their care team, and receive tailored feedback without the need for real-time interaction and have been shown to support chronic disease management and self-management, including in MS [48-50]. Our findings most likely suggest the use of asynchronous telehealth as a complementary, but not necessarily default, component of chronic MS care.

### Factors Associated With Accepting Telehealth as a Default

In our multivariable regression analysis, neither of the 3 prepostulated NASSS constructs as possible drivers of telehealth acceptance (hypotheses 1‐3) showed independent associations. For example, although prior telemedicine experience has been linked to greater acceptance in other settings, it was not significantly associated with acceptance in our cohort. Only 20% of participants reported any prior telemedicine experience—consistent with the generally low acceptance of telehealth among people living with MS in Switzerland [32]—which likely limited its influence in our analysis.

However, we found that a general acceptance toward digital communication with health care providers, as well as regular internet use to search for health care providers, was positively associated with a willingness to accept telehealth as a default. A general acceptance of digital communication may indeed ease a transition toward telehealth, accelerated by the COVID-19 pandemic, when tools for digital communication through voice and video became more widely adopted, also among older populations [8,10,29,51]. The positive association of prior experience with online searches of health care providers is more challenging to interpret but may signal a general interest in using the internet as a tool for personal health management [7,11]. In the first digital survey, frequent online searches for health care providers distinguished a subgroup of people living with MS with greater health awareness who regularly used digital tools for managing their condition [37]. In general, individuals familiar with digital tools may perceive a lower cognitive and practical barrier in using telehealth [52]. This observation could also be true for those who use the web to search for care providers. Most likely explaining the positive association between telehealth and prior use of the internet for health care provider search found in our study, because the user’s perceived effort with technology will be relatively low [45].

Individuals with SPMS were less likely to endorse telehealth as a default. This may reflect the greater physical and cognitive burden associated with SPMS—which can limit mobility, manual dexterity, and cognitive processing—and thereby reduce patients’ ability or willingness to use the internet-based devices for medical consultations [23,41].

In Switzerland, telehealth expanded during the COVID-19 pandemic [8]. Remote consultations (mainly telephone and video) are reimbursed via existing tariff positions, and some basic insurance products use telemedicine “gatekeeper” models that require initial contact with a telemedicine provider. Despite this structural role, data on outpatient MS telehealth use remain limited, and our findings suggest that people living with MS do not yet view telehealth as an acceptable default for specialist care.

### Contextualizing Our Findings Using the NASSS Framework

We further applied the NASSS framework to our findings to contextualize the results and to understand the complexity of implementing telehealth as a default in MS care [27]. Our review of the results identified four NASSS domains that are reflected by our study as follows:

1. Condition (MS complexity): MS is a heterogeneous disease with varying degrees of physical and cognitive impairment. Our finding of a negative association between SPMS and telehealth acceptance suggests that those with more severe or chronic forms of MS are less likely to accept telehealth by default, which is consistent with the literature [23,26,41].
2. Technology (telehealth use): the acceptance of telehealth as a default depends on prior digital literacy and technology exposure. In our cohort, only 20% had prior telemedicine experience, and over half expressed concerns about data security. These are moderating factors in digital health acceptance [44]. While global health bodies and the Swiss federal government promote digital health, acceptance may likely depend on how people living with MS engage with technology [1,32].
3. Value proposition (perceived benefits of telehealth)*:* over 70% of participants supported digital access to health data, but 80% declined telehealth as a default. Therefore, while most survey respondents are generally open toward provider-facing digital health tools in MS care, they seem more reluctant to actively engage with digital health technologies themselves. This finding could highlight a need to better communicate the potential benefits of telehealth to people living with MS [24,45]. While people with chronic diseases may accept certain aspects of digital health care [23], efforts are needed to better inform people living with MS on the value of telehealth to their health care.
4. Adopter system (integration into existing health care services)*:* one-third of participants who declined telehealth by default expressed the wish for support to use computer hardware or software. The presence of structured support systems is a critical factor in technology acceptance [7,44]. Addressing digital literacy gaps, easing the onboarding of persons with MS onto telehealth, and providing ongoing technical support and assistance could enhance the feasibility of telehealth as a default option for people living with MS [7,18].

While our analysis focused on 4 NASSS domains, we did not capture factors related to the organizational context, wider system, or long-term adaptation (Multimedia Appendix 4). This limits our understanding of how systemic factors—such as provider readiness, policy frameworks, and reimbursement models—may influence the acceptance of telehealth by default. These omissions are important, as they likely shape how people living with MS perceive and engage with telehealth beyond individual factors. Future research should prioritize collecting data on these broader domains to enable a more comprehensive use of the NASSS framework.

### Strengths and Limitations

Our study has several limitations. First, given our recruitment methods and mode of survey delivery, our study population is not intended to be representative of the Swiss population of persons with MS at large (details provided in Iaquinto et al )[53]. Overall, our study sample seems to be older and more frequently of a PPMS or SPMS type. Therefore, we may have underestimated the general acceptability of telehealth (even after bias mitigation through reweighting our estimates based on our earlier, more inclusive digital health survey). However, the deviations of our (reweighted) samples from the Swiss population of persons with MS are unlikely to materially alter our main conclusion that the general openness to accept telehealth is presently quite low. Second, because the survey was administered online and focused on eHealth, it is possible that more digitally interested people living with MS were more likely to participate, which could in principle bias acceptance estimates upward, although the very low observed acceptance makes substantial overestimation unlikely.

Furthermore, since our study is a secondary data analysis, omitting some NASSS-relevant items, we were unable to assess certain NASSS domains, such as organizational factors, the wider health care system, and long-term sustainability. Also, within the value proposition and adopter system domains, we were unable to consider the perspectives of health care providers and institutions. However, by assessing the perspective of people living with MS, our study offers important insights into the real-world acceptance of technology by the population of interest. Lastly, this study was conducted post-COVID-19 era, when the pressure to use telehealth is relatively low, and there is an increasing need for things to return to how they were before the pandemic, a possible reason for the low uptake.

However, our study has the following strengths. To our knowledge, this is the first study to specifically assess the acceptance of telehealth as a default in MS care. We also identified subgroups requiring targeted support to improve telehealth acceptance. Taken together, our study has identified some additional aspects that might pave the way toward an individualized telehealth setting suited for people with chronic diseases.

### Implications and Recommendations

The low acceptance of telehealth as a default highlights the need for measures that address the barriers affecting acceptance. These measures may include targeted communication campaigns or having technical staff available to assist people living with MS with technical questions during onboarding. Our findings suggest that individuals who already engage with digital health tools for health management-related tasks, such as provider searches, are more receptive to telehealth. This suggests that furthering positive experiences with digital communication and provider interactions may also enhance the acceptance of telehealth. Furthermore, given that approximately one-third of participants required support with digital devices, implementing technical support and training programs could improve uptake. Policymakers and health care providers should prioritize these measures to ensure equitable digital health care access for people living with MS.

Clinically, our data argue against telehealth as a universal default model for MS care. Since participants with more severe disease were less likely to accept telehealth as the default modality. A more nuanced approach could involve triaging people living with MS according to disease severity, stability, and complexity: telehealth could be preferentially offered to those with stable, less severe disease for routine follow-up and counseling, whereas individuals with higher disability, new or complex symptoms, or substantial care coordination needs would continue to receive in-person visits by default. Such a stratified approach may optimize resource use while respecting individual preferences and clinical safety.

### Conclusions

The acceptance and scaling of telehealth as a default is complex. Our study highlights the need for targeted implementation and testing of telehealth among a diverse cohort of people living with MS. These findings hint at potential considerations for the acceptance of telehealth as a default among people living with chronic diseases. Acceptance may be potentially improved if support is provided, and the value of these technologies is discussed with target patient groups. Such information should be accompanied by trust-building measures in data security and privacy, as well as offers for improving digital literacy for those in need. We conclude that acceptance of telehealth as a default needs to be targeted to the group of people living with chronic diseases most likely to benefit from it, and future research should explore provider perspectives and evaluate long-term strategies for integrating telehealth in MS care and chronic disease care.

## Supplementary material

10.2196/84447 Multimedia Appendix 1 Swiss Multiple Sclerosis Registry (SMSR) participants in our digital survey.

10.2196/84447 Multimedia Appendix 2 Use of technology and telemedicine experience.

10.2196/84447 Multimedia Appendix 3 Univariable logistic regression analysis.

10.2196/84447 Multimedia Appendix 4 Contextualizing our findings using the NASSS (Non-Adoption, Abandonment, Scale-Up, Spread, and Sustainability) framework.

10.2196/84447 Checklist 1 STROBE (Strengthening the Reporting of Observational Studies in Epidemiology) checklist.

## Abbreviations

aOR: adjusted odds ratio
CIS: clinically isolated syndrome
CVD: cardiovascular disease
HCP: health care professional
MS: multiple sclerosis
NASSS: Non-Adoption, Abandonment, Scale-Up, Spread, and Sustainability
PPMS: primary progressive multiple sclerosis
RRMS: relapsing-remitting multiple sclerosis
SMSR: Swiss Multiple Sclerosis Registry
SPMS: secondary progressive multiple sclerosis
STROBE: Strengthening the Reporting of Observational Studies in Epidemiology
