# Supplementary material for: Acceptability of Telehealth as the Default Modality for Multiple Sclerosis Care in Switzerland: Cross-Sectional Study
Source: JMIR Mhealth Uhealth. 2026 Jan 23;14:e84447. doi: 10.2196/84447 (PMC12829899; doi:10.2196/84447)
Supplement: Multimedia Appendix 1 [file mhealth-v14-e84447-s001.docx]

**Appendix 1: Swiss Multiple Sclerosis Registry (SMSR) participants in our digital survey.**

**Supplementary table 1-1: Swiss Multiple Sclerosis Registry (SMSR) participants in our digital survey.**

| Variables | Projection* | SMSR not included in Survey 1, with aggregation to match projection | SMSR not included in Survey 1 | Survey 1, with aggregation to match projection | SMSR survey 1 (2021) | Survey 2, with aggregation to match projection | SMSR survey 2  (2023) |
| --- | --- | --- | --- | --- | --- | --- | --- |
| Male sex | 27.5% | 27.1% | 433/1599 (27.1) | 25.7% | 254/990 (25.7) | 30.4% | 131/431 (30.4) |
| 18 - 30 years | 53% | 50% | 98/1599 (6.1) | 44.4% | 33/974 (3.4) | 40.9% | 11/431 (2.6) |
| 31 - 40 years |  |  | 317/1599 (19.8) |  | 165/974 (16.9) |  | 56/431 (13.0) |
| 41 - 50 years |  |  | 385/1599 (24.1) |  | 234/974 (24.0) |  | 109/431 (25.3) |
| 51 - 60 years | 47% | 50% | 411/1599 (25.7) | 55.6% | 305/974 (31.3) | 59.1% | 143/431 (33.2) |
| 61 - 70 years |  |  | 239/1599 (14.9) |  | 173/974 (17.8) |  | 82/431 (19.0) |
| 70+ years |  |  | 149/1599 (9.3) |  | 64/974 (6.6) |  | 30/431 (7.0) |
| Clinically isolated syndrome (CIS) or relapsing-remitting MS (RRMS) | 81.5% | 72.5% | 998/1599 (61.2) | 69.4% | 648/990 (65.4) | 66.4% | 286/431 (66.4) |
| Primary progressive MS (PPMS) | 7.5% | 12.4% | 162/1599 (10.1) | 11.8% | 108/990 (10.9) | 12.3% | 53/431 (12.3) |
| Secondary progressive MS (SPMS) | 11% | 15.1% | 198/1599 (12.4) | 18.8% | 171/990 (17.3) | 21.4% | 92/431 (21.4) |

***Adapted from Iaquinto et al, Neuroepidemiology, 2024[58]**

- *Column 1 shows the projections of people living with MS in Switzerland in 2021, based on Iaquinto et al, Neuroepidemiology, 2024.*
- *Columns 3 and 4 show the proportion of people living with MS who are part of the MS Registry, but who did not complete the first survey on digitalization (performed in three languages and paper-and-pencil, as well as online).*
- *Columns 5 and 6 show the proportion of people living with MS captured during the first survey in 2020.*
- *Column 4 is the aggregation across the same age groups as in the nationwide projection in column 1 for better comparability.*
- *Columns 6 and 7 show the proportion of people living with MS captured during the second survey in 2.*
- *Column 6 is the aggregation across the same age groups as in the nationwide projection in column 1 for better comparability*
