# Supplementary material for: Acceptability of Telehealth as the Default Modality for Multiple Sclerosis Care in Switzerland: Cross-Sectional Study
Source: JMIR Mhealth Uhealth. 2026 Jan 23;14:e84447. doi: 10.2196/84447 (PMC12829899; doi:10.2196/84447)
Supplement: Multimedia Appendix 2 [file mhealth-v14-e84447-s002.docx]

## **Appendix 2: Use of technology and telemedicine experience.**

Supplementary Table 1-2 presents participants' digital engagement, stratified by their experience with telemedicine. Overall, 335/427 (78.5%) found default digital access to health data beneficial. Digital document exchange was considered beneficial by 236/427 (55.3%) of respondents, and 167/427 (39.1%) agreed that digital communication with healthcare providers would be advantageous.

We assessed participants’ knowledge and concerns about the use of technology for health. Here, 252/427 (59.0 %) expressed concerns about data validity, and 271/427 (63.5%) cited data security concerns. Referring to concerns about the health effects of internet use, 19/427 (4.5%) participants with telemedicine experience had concerns compared to 51/427 (11.9) those without experience.

In assessing other factors that influence the use of technology for health, we looked at participants’ engagement in health-related online activities on at least a monthly basis. The most common activities were general health information search, 249/427 (58.3%) and MS-specific information search, 195/427 (45.7%). Communication with healthcare providers was reported by 57/427 (13.3%) of respondents, while only 43/427 (10.1%) engaged in self-tracking activities.

**Supplementary Table 1-2: Comparison of Telemedicine Experience**

| Variable | Overall  N (%) | No Knowledge  No Experience  N (%) | Knowledge  No Experience  N (%) | Knowledge and  Experience  N (%) |
| --- | --- | --- | --- | --- |
| N | 427 | 169 | 169 | 89 |
| Would you find any of the following functionalities beneficial for your medical care? | | | | |
| Telehealth as a default (agree). | 66(15.5) | 26 (15.4) | 26 (15.4) | 14 (15.7) |
| Sell your health data (agree). | 102 (23.9) | 41 (24.3) | 39 (23.1) | 22 (24.7) |
| Default digital access to health data (agree). | 335 (78.5) | 120 (70.0) | 140 (82.8) | 75 (84.3) |
| Default digital document exchange (agree). | 236 (55.3) | 83 (49.1) | 95 (56.2) | 58 (65.2) |
| Default digital communication with a healthcare provider (agree). | 167 (39.1) | 50 (29.6) | 75 (44.4) | 42 (47.2) |
| Which concerns do you have related to internet use? | | | | |
| Health effects of internet use | | | | |
| Agree | 70 (16.4) | 25 (14.8) | 26 (15.4) | 19 (21.3) |
| Isolation from real life | | | | |
| Agree | 97 (22.7) | 44 (26.0) | 31 (18.3) | 22 (24.7) |
| Concern about invalid information | | | | |
| Agree | 252 (59.0) | 93 (55.0) | 104 (61.5) | 55 (61.8) |
| Concerned about data security | | | | |
| Agree | 271 (63.5) | 106 (62.7) | 112 (66.3) | 53 (59.6) |
| For which activity do you go online when related to the topic of medicine and health (components for use score)? Percentage with at least one monthly activity. | | | | |
| Exchange with other patients | 41 (9.6) | 15 (8.9) | 15 (8.9) | 11 (12.4) |
| Self-tracking | 43 (10.1) | 12 (7.1) | 20 (11.8) | 11 (12.4) |
| Communication with a Healthcare provider | 57 (13.3) | 24 (14.2) | 13 (7.7) | 20 (22.5) |
| Healthcare provider search | 30 (7.0) | 15 (8.9) | 9 (5.3) | 6 (6.7) |
| MS information search | 195 (45.7) | 75 (44.4) | 78 (46.2) | 42 (47.2) |
| Health information search | 249 (58.3) | 80 (47.3) | 114 (67.5) | 55 (61.8) |
| Private appointments | 250 (58.5) | 93 (55.0) | 102 (60.4) | 55 (61.8) |
| Professional appointments | 188 (44.0) | 59 (34.9) | 80 (47.3) | 49 (55.1) |
| All appointments | 299 (70.0) | 108 (63.9) | 124 (73.4) | 67 (75.3) |
| How often do you use electronic devices (at least monthly) | | | | |
| Frequency of smartwatch use | 57 (13.3) | 16 (9.5) | 26 (15.4) | 15 (16.9) |
| Frequency of smartphone use | 365 (85.5) | 138 (81.7) | 146 (86.4) | 81 (91.0) |
| Frequency of tablet use | 146 (34.2) | 56 (33.1) | 63 (37.3) | 27 (30.3) |
| Frequency of pc use | 294 (68.9) | 108 (63.9) | 123 (72.8) | 63 (70.8) |
| In which areas do you wish for more support in the “digital” world? | | | | |
| Need support when using the internet | 104 (24.4) | 51 (30.2) | 35 (20.7) | 18 (20.2) |
| Need support when using hardware | 109 (25.5) | 48 (28.4) | 42 (24.9) | 19 (21.3) |
| Needs support for both | 129 (30.2) | 56 (33.1) | 49 (29.0) | 24 (27.0) |
