# Supplementary material for: Acceptability of Telehealth as the Default Modality for Multiple Sclerosis Care in Switzerland: Cross-Sectional Study
Source: JMIR Mhealth Uhealth. 2026 Jan 23;14:e84447. doi: 10.2196/84447 (PMC12829899; doi:10.2196/84447)
Supplement: Multimedia Appendix 3 [file mhealth-v14-e84447-s003.docx]

## **Appendix 3: Univariable logistic regression analysis**

In the univariable logistic regression analysis (Supplementary table 1-3), participants who preferred default communication with healthcare providers (HCPs) had significantly higher odds (OR: 8.83, 95% CI: 4.77 – 17.51). Also, the agreement to default digital document exchange was significantly associated (OR: 2.64, 95% CI: 1.49–4.87). Additionally, concerns about data validity (OR=0.57, CI: 0.34 – 0.97), internet use for MS information search (OR: 1.84, 95% CI: 1.06–3.23), and internet use for searching healthcare providers (OR: 2.68, 95% CI: 1.11–6.02) showed a significant association. Age, sex, and support needs were not significantly associated with default telehealth consultations.

**Supplementary table 1-3: Univariable logistic regression analysis for default telehealth consultations**

| Variable | No Telehealth Cons. (n%) | Yes, Telehealth Cons. (n%) | OR | 95%CI Lower | 95%CI Upper | P-Value |
| --- | --- | --- | --- | --- | --- | --- |
|  | 361(84.5%) | 66(15.5) |  |  |  |  |
| Descriptive variables |  |  |  |  |  |  |
| Sex: female (ref) |  |  |  |  |  |  |
| Sex: male | 105 (29.1%) | 24 (36.4%) | 1.39 | 0.79 | 2.4 | .24 |
| Age |  |  |  |  |  | .36 |
| Age: 18 - 30 years (ref) |  |  |  |  |  |  |
| Age:31-40 years | 48 (13.3%) | 8 (12.1%) |  |  |  |  |
| Age:41-50 years | 90 (24.9%) | 18 (27.3%) |  |  |  |  |
| Age:51-60 years | 121 (33.5%) | 22 (33.3%) |  |  |  |  |
| Age:61-70 years | 73 (20.2%) | 8 (12.1%) |  |  |  |  |
| Age: above 70 years | 20 (5.5%) | 8 (12.1%) |  |  |  |  |
| MS Type |  |  |  |  |  | .93 |
| MS Type: CIS/RRMS (ref) |  |  |  |  |  |  |
| MS type: PPMS | 44 (12.2%) | 7 (10.6%) |  |  |  |  |
| MS type: SPMS | 77 (21.3%) | 14 (21.2%) |  |  |  |  |
|  |  |  |  |  |  |  |
| Hypothesis 1: Have you ever used a telemedicine service? | | | | | | |
| Telemedicine experience (yes) | 75 (20.8%) | 14 (21.2%) | 1.03 | 0.52 | 1.91 | .94 |
|  |  |  |  |  |  |  |
| Hypothesis 2: Acceptability to digital default (would you find any of the following functionalities beneficial for your medical care?) | | | | | | |
| Default digital communication with HCP | 114 (31.6%) | 53 (80.3%) | 8.83 | 4.77 | 17.51 | <.01 |
| Default digital access to health data | 276 (76.5%) | 57 (86.4%) | 1.88 | 0.93 | 4.22 | .10 |
| Default digital document exchange | 187 (51.8%) | 49 (74.2%) | 2.64 | 1.49 | 4.87 | <.01 |
|  |  |  |  |  |  |  |
| Hypothesis 3: In which areas do you wish for more support in the “digital” world? | | | | | | |
| Needs support with hardware | 86 (23.8%) | 23 (34.8%) | 1.68 | 0.95 | 2.92 | .07 |
| Needs support using the internet | 85 (23.5%) | 19 (28.8%) | 1.28 | 0.7 | 2.27 | .41 |
| Needs support | 105 (29.1%) | 24 (36.4%) | 1.36 | 0.77 | 2.34 | .28 |
|  |  |  |  |  |  |  |
| Other potentially explanatory variables | | | | | | |
| Concern about invalid information | 220 (60.9%) | 32 (48.5%) | 0.57 | 0.34 | 0.97 | .04 |
| Concerned about data security | 235 (65.1%) | 36 (54.5%) | 0.62 | 0.36 | 1.06 | .08 |
| Uses the internet for health info search | 209 (57.9%) | 40 (60.6%) | 1.47 | 0.82 | 2.77 | .21 |
| Uses internet for MS info search | 157 (43.5%) | 38 (57.6%) | 1.84 | 1.06 | 3.23 | .03 |
| Uses the internet for provider search | 21 (5.8%) | 9 (13.6%) | 2.68 | 1.11 | 6.02 | .02 |
| Uses the internet for communication with the provider | 44 (12.2%) | 13 (19.7%) | 1.81 | 0.88 | 3.52 | .09 |
| Uses the internet for self-tracking | 32 (8.9%) | 11 (16.7%) | 2.32 | 1.05 | 4.82 | .03 |
| Uses the internet for patient exchange | 33 (9.1%) | 8 (12.1%) | 1.42 | 0.58 | 3.12 | .41 |

**Key : aOR= adjusted odds ratio, CI=confidence interval, ref = reference group, MS= multiple sclerosis, HCP= healthcare professional, CIS = Clinically isolated syndrome, RRMS = Relapsing-remitting MS, PPMS = Primary progressive MS, SPMS = Secondary Progressive MS.*
