# Supplementary material for: Acceptability of Telehealth as the Default Modality for Multiple Sclerosis Care in Switzerland: Cross-Sectional Study
Source: JMIR Mhealth Uhealth. 2026 Jan 23;14:e84447. doi: 10.2196/84447 (PMC12829899; doi:10.2196/84447)
Supplement: Multimedia Appendix 4 [file mhealth-v14-e84447-s004.docx]

## **Appendix 4: Contextualising our findings using the** **Non-Adoption, Abandonment, Scale-Up, Spread, and Sustainability (NASSS) Framework**

**Supplementary table 1-4: NASSS domains assessed in our study.**

| NASSS Domain | Question Applied in Our Context | Complexity* (Simple, Complicated, Complex) | Comment(s) |
| --- | --- | --- | --- |
| Domain 1: The Condition/Illness |  |  |  |
| 1A. What is the nature of the condition or illness? | Type of MS** | Complex | MS is a progressive and heterogeneous condition, with increasing impairment. This complexity influences the openness to telehealth as a default. |
| Domain 2: The Technology |  |  |  |
| 2C. What knowledge and/or support is required to use the technology? | Previous experience with digital devices | Complicated | While prior experience with digital devices was assessed, applying this knowledge to telehealth may be challenging, depending on disease progression and past digital health exposure. |
| Domain 3: The Value Proposition |  |  |  |
| 3B. What is its desirability, efficacy, safety, and cost-effectiveness (demand-side value)? | Perceived benefits of telehealth for people living with MS | Complicated | Telehealth may improve access to care and reduce costs for people living with MS, but clearer communication with people living with MS is needed to ensure understanding and acceptance. |
| Domain 4: The Adopter System |  |  |  |
| 4B. What is expected of the patient (and/or immediate caregiver)—and is this achievable and acceptable? | Knowledge and prior experience with telemedicine | Complicated | A significant proportion of people living with MS with prior telemedicine experience still required digital support, indicating that previous exposure alone may not ensure readiness for default telehealth consultations. |
| Domain 5: The Organization |  |  |  |
| 5A. What is the organization’s capacity to innovate? | General healthcare system openness to digital health acceptance | Complicated | While the study focused on patient perspectives, the Swiss healthcare system is generally open to digital health innovations, which could support implementation. |
| Domain 6: The Wider Context |  |  |  |
| 6A. What is the political, economic, regulatory, professional (e.g., medicolegal), and sociocultural context for program rollout? | Publicly available information on telehealth acceptance feasibility | Complicated | While not directly assessed, telehealth acceptance appears feasible within Switzerland’s healthcare system, though regulatory and financial considerations remain key factors. |

***** *Technologies can be classified as simple (few components, predictable), complicated (many components, largely predictable), or complex (many components interacting in dynamic and unpredictable ways).*

***Multiple sclerosis*

**Supplementary table 1-5: Aspects of the NASSS framework not assessed.**

| NASSS Domain | Question Applied in Our Context | Complexity* | Comment(s) |
| --- | --- | --- | --- |
| Domain 1: The Condition/Illness | 1 B. What are the relevant sociocultural factors and comorbidities? | √ | This study did not assess sociocultural factors influencing people living with MS in Switzerland. Future research could explore these dimensions. |
| Domain 2: The Technology | 2a. What are the key features of the technology? | √ | The study did not evaluate specific telehealth system features. |
| 2 B. What kind of knowledge does the technology bring into play? | √ | No explicit assessment was made on how telehealth transforms healthcare knowledge for people living with MS. |  |
| 2D. What is the technology supply model? | √ | The study did not focus on telehealth infrastructure or supply models. |  |
| Domain 3: The Value Proposition | 3A. What is the developer’s business case for the technology (supply-side value)? | √ | The study did not assess commercial incentives behind telehealth acceptance. |
| Domain 4: The Adopter System | 4A. What changes in staff roles, practices, and identities are implied? | √ | The study focused on patient experiences with telehealth rather than provider roles. |
| 4C. What is assumed about the extended network of lay caregivers? | √ | The role of caregivers in supporting telehealth use was not explored. |  |
| Domain 5: The Organization | 5 B. How ready is the organization for this technology-supported change? | √ | Institutional readiness for telehealth was not assessed. |
| 5C. How easy will the acceptance and funding decision be? | √ | The study did not explore financial or policy factors influencing telehealth acceptance. |  |
| 5D. What changes will be needed in team interactions and routines? | √ | Healthcare team workflow adjustments were beyond the study’s scope. |  |
| 5E. What work is involved in implementation, and who will do it? | √ | The study did not assess telehealth implementation strategies. |  |
| Domain 7: Embedding and Adaptation Over Time | 7A. How much scope is there for adapting and co-evolving the technology and the service over time? | √ | Long-term adaptation of telehealth services was not analyzed. |
| 7B. How resilient is the organization in handling critical events and adapting to unforeseen challenges? | √ | Institutional resilience in integrating telehealth remains an open question. |  |

***** *Technologies can be classified as simple (few components, predictable), complicated (many components, largely predictable), or complex (many components interacting in dynamic and unpredictable ways).*

***Multiple sclerosis*
